# Identification of a clonal population of *Aspergillus flavus* by MALDI-TOF mass spectrometry using deep learning

**DOI:** 10.1038/s41598-022-05647-4

**Published:** 2022-01-28

**Authors:** Anne-Cécile Normand, Aurélien Chaline, Noshine Mohammad, Alexandre Godmer, Aniss Acherar, Antoine Huguenin, Stéphane Ranque, Xavier Tannier, Renaud Piarroux

**Affiliations:** 1 AP-HP, Groupe Hospitalier Pitié-Salpêtrière, Service de Parasitologie-Mycologie, 75013 Paris, France; 2 Section Informatique et Communication, École Polytechnique Fédérale de Lausanne (EPFL), 1015 Lausanne, Switzerland; 3 Sorbonne Université, INSERM, Institut Pierre Louis d’Epidémiologie et de Santé Publique, AP-HP, Hôpital Pitié-Salpêtrière, 75013 Paris, France; 4 Département de Bactériologie, Hôpital Saint-Antoine, AP-HP, Sorbonne Université, Paris, France; 5 Centre d’Immunologie et des Maladies Infectieuses, INSERM, U1135, Sorbonne Université, Paris, France; 6 Université de Reims Champagne Ardenne, ESCAPE EA7510, 51100 Reims, France; 7 Laboratoire de Parasitologie Mycologie, Pôle de Biopathologie, CHU de Reims Hôpital Maison Blanche, 51100 Reims, France; 8 IHU-Méditerranée Infection, 13005 Marseille, France; 9 Sorbonne Université, Université Sorbonne Paris Nord, INSERM, Laboratoire d’Informatique Médicale et d’Ingénierie des connaissances en e-Santé, LIMICS, Paris, France

**Keywords:** Mass spectrometry, Data processing, Machine learning

## Abstract

The spread of fungal clones is hard to detect in the daily routines in clinical laboratories, and there is a need for new tools that can facilitate clone detection within a set of strains. Currently, Matrix Assisted Laser Desorption-Ionization Time-of-Flight Mass Spectrometry is extensively used to identify microbial isolates at the species level. Since most of clinical laboratories are equipped with this technology, there is a question of whether this equipment can sort a particular clone from a population of various isolates of the same species. We performed an experiment in which 19 clonal isolates of *Aspergillus flavus* initially collected on contaminated surgical masks were included in a set of 55 *A. flavus* isolates of various origins. A simple convolutional neural network (CNN) was trained to detect the isolates belonging to the clone. In this experiment, the training and testing sets were totally independent, and different MALDI-TOF devices (Microflex) were used for the training and testing phases. The CNN was used to correctly sort a large portion of the isolates, with excellent (> 93%) accuracy for two of the three devices used and with less accuracy for the third device (69%), which was older and needed to have the laser replaced.

## Introduction

Comparing isolates to determine whether they are derived from the same clone is of upmost interest to identify outbreaks or reveal laboratory contaminations^1^. This process also helps to elucidate transmission patterns by linking clinical isolates to each other or to environmental isolates^2–4^.

Several approaches have been proposed for isolate matching. Historically, isolates were typed based on phenotypic methods or nonexact molecular methods such as restriction fragment length polymorphism (RFLP) or random amplified polymorphic DNA (RAPD)^1,5^. Due to their poor performance and reproducibility, these methods have been abandoned in favor of more recent exact molecular methods: microsatellite length polymorphism (MLP), also known as short tandem repeats (STR), multilocus sequence typing (MLST), and more recently next-generation sequencing (NGS). These techniques have excellent discriminatory power and reproducible results. Moreover, MLST can be used to build globally shared databases. Whole-genome sequencing is also an interesting approach, but it remains too expensive and time consuming to be implemented in routine clinical microbiology experiments^6^.

A main disadvantage of all these approaches is the requirement of extra experimental procedures, in addition to routine species identification, each time a new isolate is collected. In the daily activities of a clinical mycology laboratory, isolates are generally collected over time as the outbreak is investigated. Comparing these isolates with already collected isolates belonging to a given clone requires the implementation of extra typing procedures that consume resources and time. This additional workflow is not suitable to incorporate into the routine activities of clinical laboratories. A tool enabling the detection of clonal populations in a quick and easy manner using a technique that is already systematically performed on each of the isolates would be invaluable during an outbreak.

Currently, routine identification of human, animal and plant pathogenic fungi uses MALDI-TOF-based assays. This method produces a protein profile, considered a species-specific fingerprint of the microorganism, using mass spectrometry. This technique permits high-throughput identification of a wide range of microorganisms by comparing their mass spectra with reference spectra included in a database. In recent years, MALDI-TOF has been shown to be more precise and more accurate than traditional identification relying on macroscopic and microscopic morphological criteria^7,8^. For instance, MALDI-TOF has the ability to properly differentiate between cryptic and sensu-stricto species of the same section of *Aspergillus*^9^.

A MALDI-TOF mass spectrum consists of a relatively complex protein profile made of hundreds of peaks that can be characterized by their mass/charge ratio (m/Z) and their intensity. If properly used, this large amount of data is likely to provide information going further than simple species identification. Several studies have emphasized the potential of artificial neural networks (ANNs) for extracting informative patterns from MALDI-TOF mass spectra in biology. For instance, Wang et al. developed machine-learning models permitting the screening for heterogeneous vancomycin-intermediate *Staphylococcus aureus*^10^ or the recognition of different MLST types of methicillin-resistant *Staphylococcus aureus*^11^. In a recent study dealing with the use of mass spectra in entomology^12^, Nabet et al*.* succeeded in extracting information that predicts age, blood-meal status and Plasmodium carriage from female Anopheles MALDI-TOF mass spectra.

In this paper, we aim to evaluate whether an ANN approach, and more precisely a convolutional neural network (CNN) approach, allows the identification of an epidemic clone in a population of isolates belonging to the same fungal species using MALDI-TOF mass spectra. We chose the real-life example of an episode of surgical mask contamination by an *Aspergillus flavus* clone that occurred in 2015–2016 in Marseille University Hospital. More specifically, we tested the ability of a neural network analysis of MALDI-TOF mass spectra to distinguish the spectra corresponding to 19 isolates, obtained from cultures of masks, and belonging to this clone among a set of spectra derived from a panel of 36 nonclonal strains of *A. flavus* from various origins (4 from other masks and 32 from patients from different hospitals).

## Methods

In 2015–2016, the laboratory of mycology at the Marseille University Hospital was commissioned to investigate the presence of mold in several batches of surgical masks with a moldy odor. These batches were from various suppliers.

### *Aspergillus flavus* culture and identification

The masks were introduced into a blender bag (Gosselin, France) and washed in 15 mL of Tween^®^ 80 at 0.1% in saline solution for 10 min in a stomacher (Homogenius HG400, Mayo International, Italy). The liquid was then transferred to a 15 mL falcon tube and centrifuged for 10 min at 3500 rpm. The supernatant was discarded, and the pellet was resuspended in 500 µL of washing solution. The subsequent 500 µL was immediately cultured on a Sabouraud-Chloramphenicol petri dish. *Aspergillus flavus* colonies were identified from 23 batches of masks using the recently released MSI-2 application (that allows the online identification of fungal MALDI-TOF mass spectra)^13^ and subjected to microsatellite typing together with 32 isolates randomly selected from the daily workflow of four French teaching hospitals (Hôpital de la Pitié Salpêtrière in Paris, CHU of Bordeaux, CHU of Toulouse, and CHU of Montpellier) to obtain a large diversity of microsatellite profiles. List and origin of the 55 isolates can be found in Supplementary Table 1.

### Microsatellite typing

All *A. flavus* isolates were typed using the protocol and microsatellites described by Hadrich et al. in 2010^14^. This publication showed that a combination of five of the twelve tested microsatellites was the most parsimonious panel, achieving a Simpson index of diversity (D) over 0.95. For our study, we used these five microsatellites: AFLA1, AFLA3, AFLA7, AFPM3 and AFPM7. However, the AFPM3 marker (a marker with a complex motif ((AT)6AAGGGCG(GA)) was dropped, as no positive PCR was obtained for 21 of the 23 isolates of *Aspergillus flavus* collected from the masks. Among the four remaining microsatellites, AFLA3, AFLA7 and AFPM7 markers could be amplified for all samples and were used to build a UPGMA tree that included all 55 isolates to be tested. UPGMA tree was built using the http://genomes.urv.cat/UPGMA/ website and considering our data as categorical values. For 10 isolates from the panel of patient isolates, the AFLA1 marker could not be read; hence, we used the value of this AFLA1 marker only to confirm clonality and strengthen the clone definition of the isolates. Simpson index of diversity using three (AFLA3–AFLA7–AFPM7) or four (AFLA3–AFLA7–AFPM7–AFLA1) markers used here are almost equal to 0.88 with our data.

### MALDI-TOF MS protein extraction

Each isolate of *A. flavus* was first thawed as they had been stored in preserving liquid with ceramic bids at − 80 °C for up to 3 years. One or two bids were used for culture on Sabouraud chloramphenicol gentamicin agar (SCG) and incubated for one week at 30 °C to obtain sporulating isolates. After this incubation period, each isolate was subcultured on a new SCG plate and incubated for three (“Day-3”) and five (“Day-5”) days at 30 °C. This subculture in two steps allowed similar growth for all isolates, as some of them took more time to be revived. At the two ages of culture, protein extraction using consecutive formic acid and acetonitrile incubations was performed as previously described^15^.

### Workflow for MS acquisition

**Figure 1:**
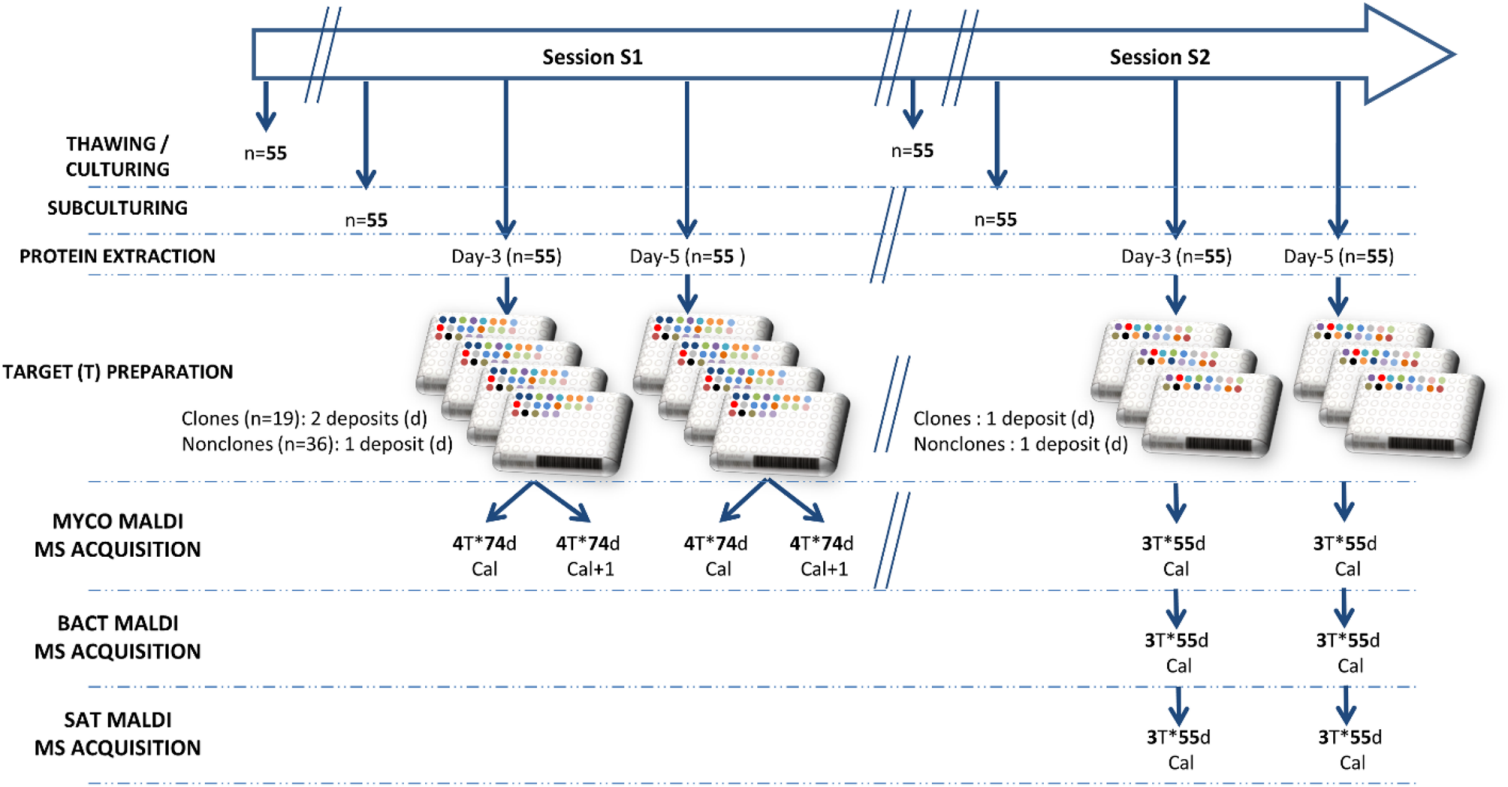
Culture and spectral acquisition workflow for both training (S1) and testing (S2) phases. T = Target, d = deposit, Cal = Microflex calibration of the day, Cal + 1 = Microflex calibration of the day after. In the MS Acquisition lines, 4 T*74d means that 74 deposits were made on 4 Targets. MYCO (mycology department), BACT (bacteriology department) and SAT (Saint Antoine hospital) correspond to the laboratories where the three MALDI TOF instrument used for this study are located.

The 55 isolates were cultivated twice, at a one-month delay, in the same culture conditions, on SCG culture media (Oxoid, Dardilly, France). The first cultivation batch was used for the training process of the CNN, while the second cultivation batch was used only for testing. The workflow is schematized in Fig. 1.

After determination of the microsatellite profile of each of the 55 isolates and protein extraction of all isolates, the number of clonal and nonclonal deposits were equilibrated for the training part of the study (Session S1). To this end, the protein extracts from clones were deposited on two spots on the MALDI-TOF MS target, while the other extracts were only deposited on one spot. The 55 isolates were deposited on the same target. To multiply the number of available spectra for training, four targets were prepared in parallel, and spectra acquisition was performed again the next day, using a new calibration of the Microflex.

The second session of spectral acquisitions (S2) aimed at testing the neural network developed from the training session (S1). Here, we deposited the protein extract on only one spot per isolate on the target, as would be done under routine conditions. However, deposits were performed on three targets that were evaluated on three different Microflex mass spectrometers (one in the mycology laboratory of the Saint-Antoine Hospital in Paris (SAT-MS), one in the mycology laboratory (MYCO-MS: the same one as for the learning phase) and one in the bacteriology laboratory of the Pitié Salpêtrière hospital (BACT-MS)). Our goal was to test for the robustness of the results from one device to another.

### Selection of the spectra for training versus testing

To increase the statistical reliability, we performed a cross-validation of the CNN with iterative experiments (n = 30) in which 44 isolates (80% of the isolates) were randomly selected to train a neural network with corresponding mass spectra obtained during the first session (S1) of culture. For each of the 30 iterations, the mass spectra corresponding to the 11 remaining isolates (20% of the isolates) were ignored to avoid sharing isolates in the training and testing sections. Instead, for those 11 isolates, spectra were selected from the second session (S2) of cultures obtained one month after the first culture session and were included in the testing set. After every iteration of training/testing, the training data were reset, and a new random selection of 44 isolates for training using the S1 spectra and of 11 isolates for testing using the S2 spectra was performed. Selection of the isolates for training/testing was performed following a stratified random sampling method. Each training set has approximately 980 spectra, and each testing set has 22 spectra.

For each experiment, the sets of mass spectra used for training and for testing were obtained from cultures, and mass spectra acquisitions were performed one month apart. The two different culture ages were pooled for the training phase, while they were analyzed either separately or pooled for testing of the neural network. Furthermore, while we used a single mass spectrometer to acquire the spectra used for training, we acquired the spectra of the isolates allocated to the testing on three devices. The goal was to check whether a neural network trained on one device maintained the ability to identify additional isolates cultured at a different time and with spectra acquired on another device.

### Convolutional neural network (CNN): system architecture and training process

**Figure 2:**
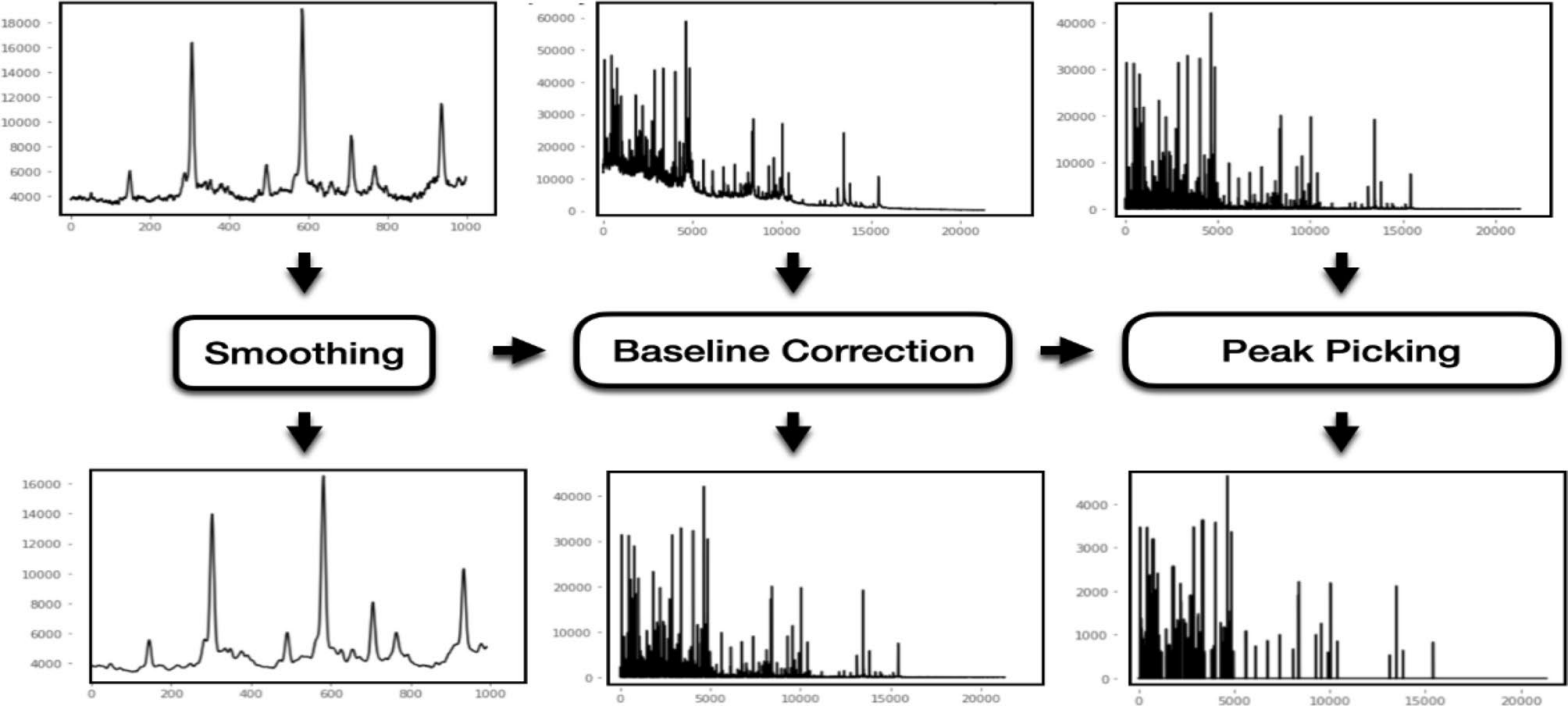
Schematization of the three-step preprocessing treatment of the spectra with the baseline subtraction, the Fourier transformation, and the peak picking. x-axis = m/Z; y-axis = intensity.

Raw data extracted from the fid files of each of the spectra were treated the same way prior to being included in the CNN. In all, approximately 22,000 intensity values (corresponding to 22,000 m/Z) composing a spectrum were submitted to a three-step preprocessing. First, the baseline was subtracted using the asymmetric least squares smoothing method^16^. Second, a Fourier transformation was applied to smooth the spectra to avoid small variations in intensity. Third, peaks were picked by computing the derivative of the intensity and then detecting the sign changes in the spectra derivative^17^. Treatment of the spectra is schematized in Fig. 2.

A CNN is a special type of deep learning model for processing data that has a grid pattern and is designed to automatically and adaptively learn spatial hierarchies of features from low- to high-level patterns. In our case, we work with a 1D CNN, as the spectra are represented one-dimensionally. The idea is to see if the CNNs manage to read spectra as images and capture local patterns that are peaks of interest and thus manage to classify them. As a first intention for testing a neural network on fungal mass spectra, we constructed a simple CNN using TensorFlow v2.0.0.

The CNN model contains 1 input layer, 1 convolutional layer, 1 max pooling layer, 1 Flatten layer, 1 fully connected layer, and 1 softmax^18^ layer to form the output prediction. The number of filters and the kernel size were set as 8 and 16, respectively, for the convolutional layer. The activation function of the convolutional layer and the fully connected layer were rectified linear units (ReLUs). The softmax function was applied in the last layer to produce the prediction probability over the two output classes: Clones (CL) and Non-Clones (NC).

**Figure 3:**
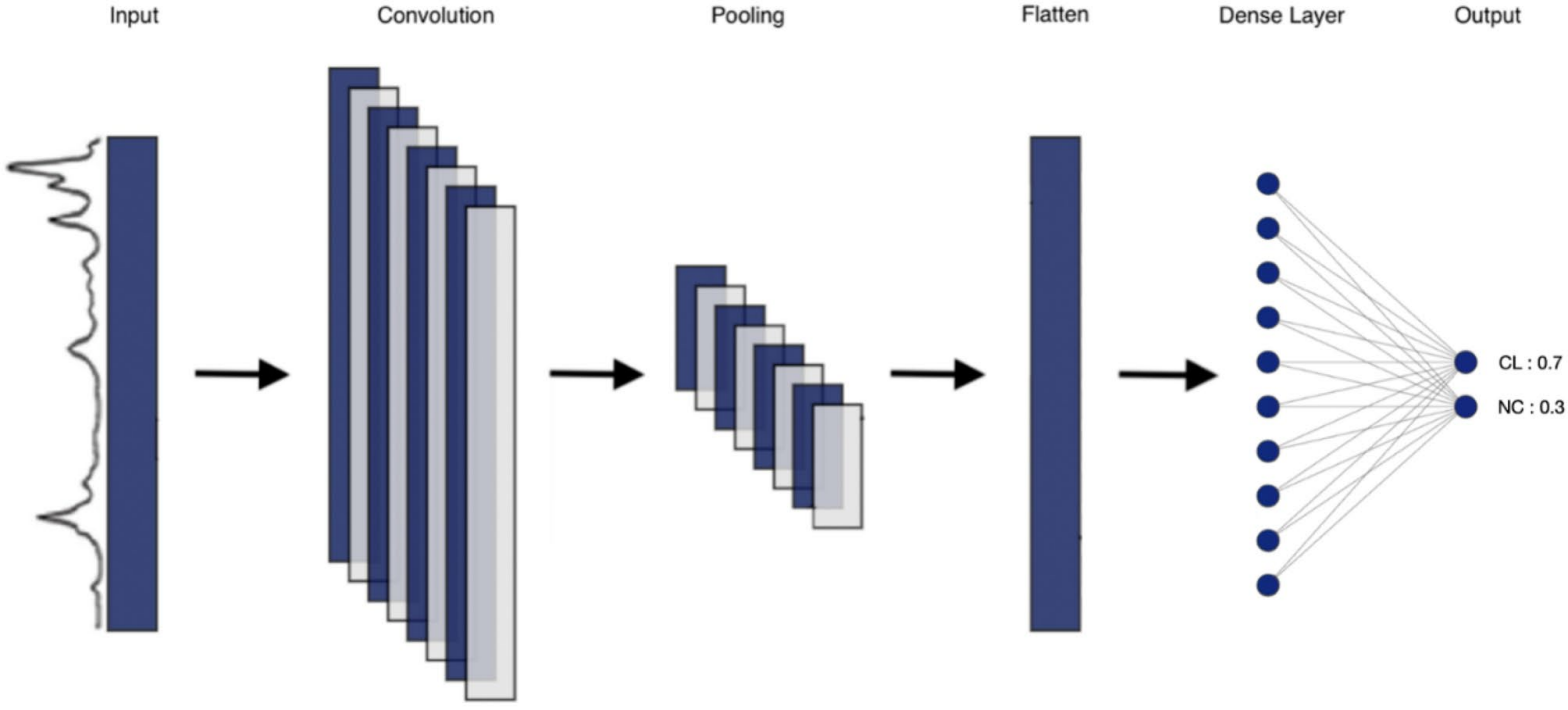
Schematization of the CNN model used for this experiment. The 6 layers are represented (input layer, convolutional layer, max pooling layer, Flatten layer, fully connected layer, and softmax layer). There are two output classes: Clones (CL) and Nonclones (NC). The sum of the predictions for each output classes is always equal to 1.

Categorical cross-entropy was selected as the loss function, and the Adam algorithm was selected as the optimizer. The learning rate was set at 0.001, and the number of epochs was set at 100. A schematization of this CNN can be found in Fig. 3.

### Statistical analyzes

For each spectrum submitted to the CNN, the result is expressed as a prediction (probability that the spectrum corresponds to each of the output classes), and the probability that a spectrum corresponds to a clone plus the probability that it corresponds to a nonclone always equals one. The accuracy (percentage of correct identifications), precision (proportion of true positive categorizations among the positive categorizations = positive predictive value) and recall (capacity to identify the clones = sensitivity) for the 30 iterations are calculated and compiled to assess the CNN.

## Results

### Determination of the clonality of the isolates by microsatellite analysis

**Figure 4:**
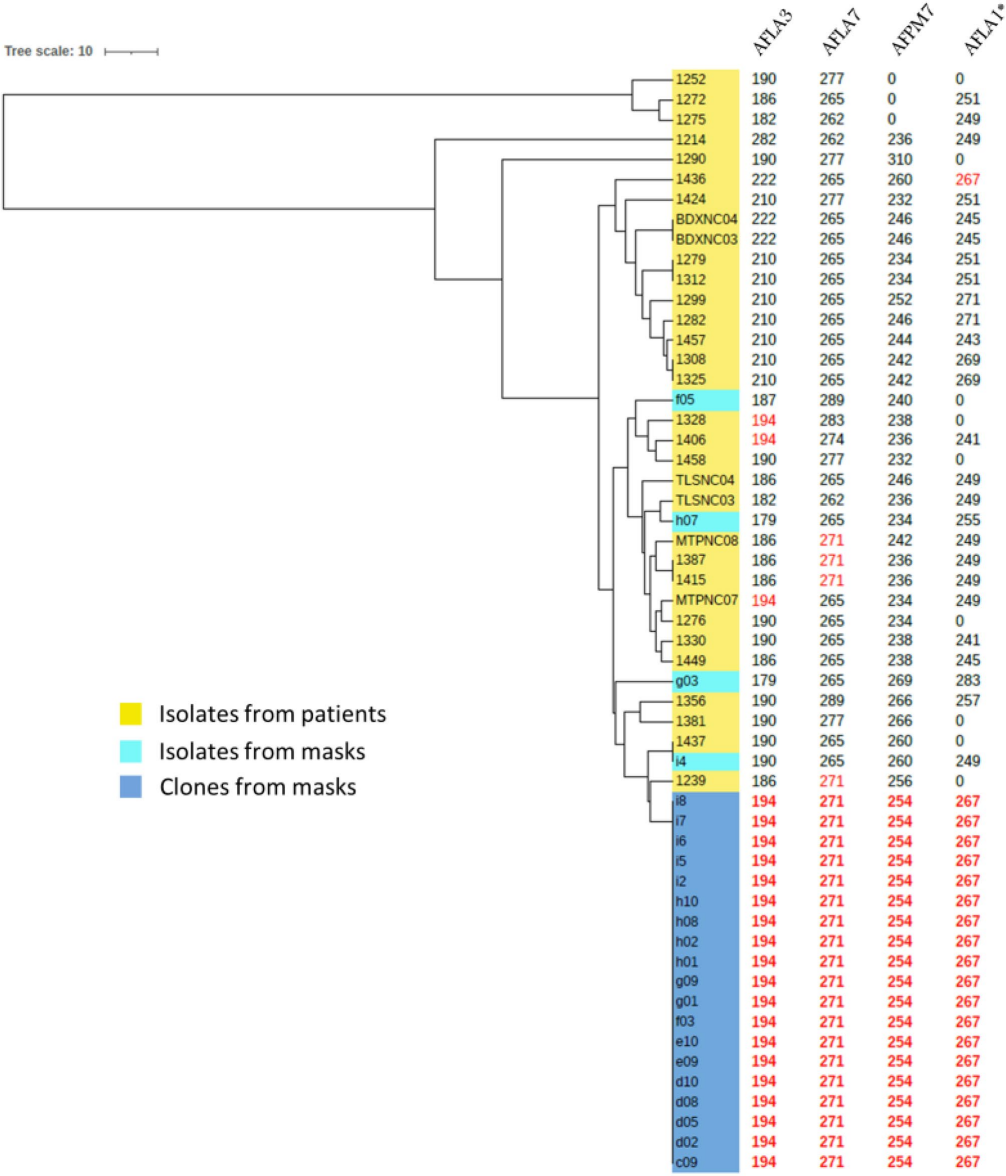
UPGMA tree using the three microsatellites that were positive for all isolates (AFLA3, AFLA7 and AFMP7). Size values for all microsatellites are indicated in columns. *AFLA1 values are given as an indicator, but were not used to build the tree. Values in red correspond to alleles sizes that are identical to the allele size of the clone.

Among the 55 tested isolates, 19 of the 23 isolates from masks showed the same microsatellite pattern and clustered into a unique branch by the microsatellite analysis (Fig. 4). The four remaining isolates from masks showed different microsatellite patterns and were not clonal. Otherwise, four other pairs (BDXNC03 and BDXNC04; 1312 and 1279; 1325 and 1308; 1415 and 1387) were found in the isolates from patients, but these showed different microsatellite patterns and clustered in different branches from the 19 clonal isolates from masks.

### CNN performance

**Figure 5:**
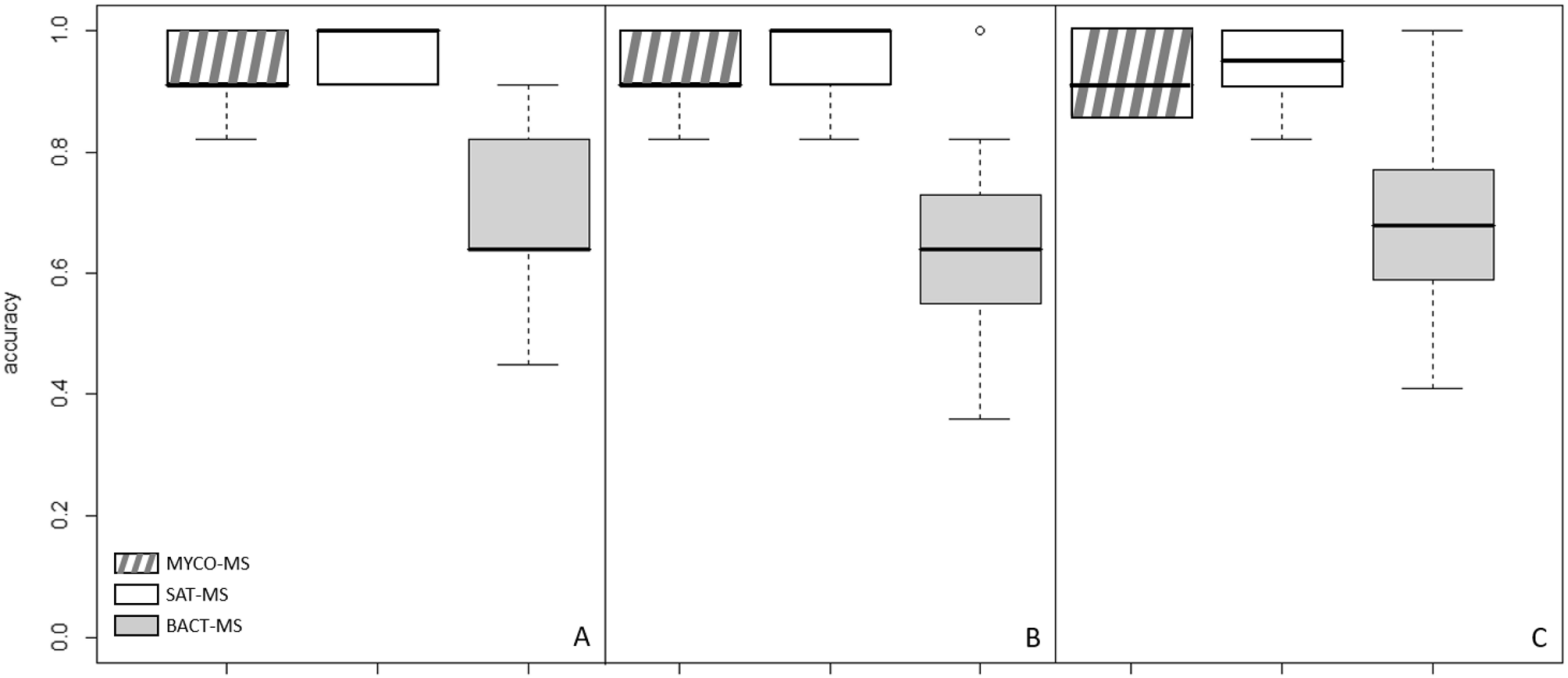
Classification capacity of the CNN model for the three different mass spectrometers. Classification results with test spectra from Day-3 (**A**), Day-5 (**B**) of growth, and global assessment using both spectra from Day-3 and Day-5 (**C**).

Figure 5 shows the classification capacity of our CNN model for the three different mass spectrometers when the training session was performed on a unique machine (MYCO-MS). Training was performed with both spectra from Day-3 and Day-5. However, we decided to evaluate the classification results with test spectra from Day-3 and Day-5 of growth separately (Fig. 5A,B, respectively). A global assessment of the classification was also performed using spectra from Day-3 and Day-5 of growth mixed together (Fig. 5C).

Spectra could be categorized with a mean accuracy of 93% at Day-3, 95% at Day-5 and 93% with the two culture times for the MYCO-MS. Mean accuracies of 96% at Day-3 and Day-5 of culture and of 94% with the two ages of culture together could be observed with spectra obtained with the SAT-MS. The mean percentage of correct identification was much lower for the BACT-MS (71% at Day-3, 65% at Day-5 of culture and 69% with the two ages together).

In this study, in addition to the accuracy, two other measurements of performance were calculated: precision and recall on the clone label. The prediction resulted in 86% mean precision at Day-3 and Day-5 of culture for the MYCO-MS and 88% and 56% for the SAT-MS and BACT-MS, respectively. Clones could be classified with a mean recall of 98% at Day-3 and Day-5 of culture for the MYCO-MS, 98% for the STA-MS and 97% for the BACT-MS. The details of the recall performances for each experiment show that the recall is almost always 100% in the Clones class. This observation means that the model predicts the Clones class more often than the Nonclones class, leading to some misclassifications that result in spectra from a nonclonal isolate being classified in the Clones class.

## Discussion

We endeavored to test the capacities of a CNN applied to MALDI-TOF MS spectra to identify a particular clone in a given population of isolates. To accomplish this, we decided to use the *A. flavus* clones that were identified in 2016 in several batches of surgical masks and try to distinguish them from other *A. flavus* isolates. This experiment mimics the contents of a fungal clone that spreads during an epidemic or as a result of laboratory contamination.

The aim of all these experiments was to best reproduce the situation in which the detection of an epidemic problem led to the training of a neural network that will be further used to detect new isolates belonging to the same epidemic clone, either in the same laboratory or in a different laboratory.

Due to the relatively low number of isolates at our disposal, we performed a cross validation of the CNN by taking eleven isolates that were kept for testing out of the independent iterative training sessions. In this way, we enhanced the number of isolates included in the testing experiments while ensuring that the isolates that were used for testing were never implied in the training session. To reproduce the timing of the search for new clonal isolates during an epidemic, the training phase and the testing phase were carried out at different times, either on the same machine (but with different settings since the machine was calibrated every day) or on a different machine in another laboratory in various Paris hospitals. Furthermore, clonal and nonclonal extracts were randomly positioned on the targets to reproduce, as best as possible, the day-to-day workflow of a mass spectrometry analysis.

Convolutional neural networks are powerful discriminative models that are only now starting to be used in the medical domain, mostly in image recognition in radiology applied to oncology^16,17^. The method is potentially more acute than anything a human brain could accomplish, as it is able to assess and remember a larger number of data with small variations; hence, our desire to test it with the differentiation of mass spectra very similar to one another, all identified as belonging to the same species. Here, we tested a simple single layer CNN, and we showed that it produces promising classification scores, even though improvement can be made, especially regarding the results obtained with the BACT-MS. Our results were obtained with a limited number of isolates belonging to the clone category (19 which were compared to a panel of 36 isolates from various origins). In some cases, lowering this number would be of interest but we do not have enough evidence yet to define a lower limit of isolates that would be necessary to obtain satisfactory machine learning results. One of the limits of using CNNs is that they are highly dependent on the quality of the data and thus the available MS machine. Due to the lower accuracy observed with the BACT-MS, we look in detail at the spectra to visualize the differences between the machines (Supplementary Fig. 1) and we examined the machine parameters. Peaks in the lower masses lacked in intensity for both clones and non-clone with the BACT-MS compared to the two other instruments. The calibration of the three machines was comparable, but we noticed that the laser in the BACT-MS machine was older (6 years old compared to 2 and 3 years old for the other machines) and that it had already been used for a larger number of shots (14,179,000 for the SAT-MS, 24,042,000 for the MYCO-MS and 97,705,000 for the BACT-MS). Indeed, it is of great interest to test the model on data acquired on nonlocal mass spectrometers, as it will allow the globalization of the method. It would also be interesting to expand the current model or use improved models to identify clones of other species that may help in preventing epidemic issues, as MALDI-TOF identifications are now the first line of identifications in clinical laboratories.

Nevertheless, this simple CNN needs further improvements to better ensure the clone identification task, particularly when spectra exhibit only small variations in mass and intensity. Differences in machine tunings due to local technical configurations or due to aging of the various components of the machine can lead to a reduction in the classification performance, as observed in our study. Nevertheless, we showed here encouraging results regarding the distinction of clonal and nonclonal isolates using a set of spectra obtained from different MS machines. Other neural networks, especially deeper CNNs, Siamese CNNs, recurrent neural networks (RNNs) or hybrid neural networks, should be tested because they might give more reliable and reproducible classification results by freeing oneself from the local variations linked to the machine environment. To apply the model to other biological purposes, it will be important to build applications capable of detecting the presence of a clone already characterized from the mass spectra generated by the routine activity of laboratories. Online identification systems, as is the case for the former MSI^19^ and the new MSI-2^13^ applications used for the precise identification of fungi involved in human and animal pathology, could be an asset by making it possible to search for a clone not only in the laboratory that first reported the epidemic phenomenon and provided spectra to train a neural network but also for all the other laboratories that add their spectra to the identification system.

## Supplementary Information

Supplementary Information.

## References

[1] Alanio A, Desnos-Ollivier M, Garcia-Hermoso D, Bretagne S (2017). Investigating clinical issues by genotyping of medically important fungi: Why and how?. Clin. Microbiol. Rev..

[2] Martini C (2020). Prevalence and clonal distribution of azole-resistant *Candida parapsilosis* isolates causing bloodstream infections in a large Italian Hospital. Front. Cell. Infect. Microbiol..

[3] Gheith S (2016). Microsatellite typing of *Aspergillus flavus* strains in a Tunisian onco-hematology unit. Mycopathologia.

[4] Fekkar A (2021). Hospital outbreak of fluconazole-resistant *Candida parapsilosis*: Arguments for clonal transmission and long-term persistence. Antimicrob. Agents Chemother..

[5] Hunter PR (1991). A critical review of typing methods for *Candida albicans* and their applications. Crit. Rev. Microbiol..

[6] Gilchrist CA, Turner SD, Riley MF, Petri WA, Hewlett EL (2015). Whole-genome sequencing in outbreak analysis. Clin. Microbiol. Rev..

[7] Patel R (2019). A Moldy application of MALDI: MALDI-ToF mass spectrometry for fungal identification. J. Fungi.

[8] Gautier M (2014). Matrix-assisted laser desorption ionization time-of-flight mass spectrometry: Revolutionizing clinical laboratory diagnosis of mould infections. Clin. Microbiol. Infect..

[9] Imbert S (2019). Multi-centric evaluation of the online MSI platform for the identification of cryptic and rare species of Aspergillus by MALDI-TOF. Med. Mycol..

[10] Wang H-Y (2018). Rapid detection of heterogeneous vancomycin-intermediate *Staphylococcus aureus* based on matrix-assisted laser desorption ionization time-of-flight: Using a machine learning approach and unbiased validation. Front. Microbiol..

[11] Wang H-Y (2018). A new scheme for strain typing of methicillin-resistant *Staphylococcus aureus* on the basis of matrix-assisted laser desorption ionization time-of-flight mass spectrometry by using machine learning approach. PLoS One.

[12] Nabet C (2020). Prediction of malaria transmission drivers in Anopheles mosquitoes using artificial intelligence coupled to MALDI-TOF mass spectrometry. Sci. Rep..

[13] Normand, A.-C. *et al.* Identification of molds with MALDI-TOF mass spectrometry: Performance of the newly developed MSI-2 application in comparison with the Bruker filamentous fungi database and MSI-1. *J. Clin. Microbiol.* **0**, JCM.01299-21 (2021). 10.1128/JCM.01299-21 PMC8451417 34319807

[14] Hadrich I, Makni F, Ayadi A, Ranque S (2010). Microsatellite typing to trace Aspergillus flavus infections in a hematology unit. J. Clin. Microbiol..

[15] Cassagne C (2011). Mould routine identification in the clinical laboratory by matrix-assisted laser desorption ionization time-of-flight mass spectrometry. PLoS One.

[16] Eilers PH, Boelens HF (2005). Baseline correction with asymmetric least squares smoothing. Leiden Univ. Med. Cent. Rep..

[17] He QP, Wang J, Mobley JA, Richman J, Grizzle WE (2011). Self-calibrated warping for mass spectra alignment. Cancer Inform..

[18] Bridle, J. S. Training stochastic model recognition algorithms as networks can lead to maximum mutual information estimation of parameters. In *NIPS’89* 211–217 (1989).

[19] Normand AC (2017). Validation of a new web application for identification of fungi by use of matrix-assisted laser desorption ionization-time of flight mass spectrometry. J. Clin. Microbiol..

