# Supplementary material for: Identification of a clonal population of Aspergillus flavus by MALDI-TOF mass spectrometry using deep learning
Source: Sci Rep. 2022 Jan 28;12:1575. doi: 10.1038/s41598-022-05647-4 (PMC8799650; doi:10.1038/s41598-022-05647-4)

Supplementary Informations:

Identification of a clonal population of *Aspergillus flavus* by MALDI-TOF mass spectrometry using deep learning.

Anne-Cécile Normand^1*^, Aurélien Chaline^1,2^, Noshine Mohammad^3^, Alexandre Godmer^4,5^**,** Aniss Acherar^3^, Antoine Huguenin^6,7^, Stéphane Ranque^8^, Xavier Tannier^9^, Renaud Piarroux^1,3^

Supplemental table 1: list and origin of the 55 *Aspergillus flavus* isolates selected for this study.

|  | code | sampling date | city | mask model |  |  |
| --- | --- | --- | --- | --- | --- | --- |
| MASKS | c09 | November 2015 | Marseille | MEDLINE NON27410 |  |  |
|  | d02 | November 2015 | Marseille | CAP2B CA-DIFF |  |  |
|  | d05 | August 2015 | Marseille | KC AM4140362 |  |  |
|  | d08 | May 2015 | Marseille | KC AM5083421 |  |  |
|  | d10 | November 2015 | Marseille | CAP2B CA-DIFF |  |  |
|  | e09 | August 2015 | Marseille | KC AM5084421 |  |  |
|  | e10 | August 2015 | Marseille | KC AM5083421 |  |  |
|  | f03 | November 2015 | Marseille | MEDLINE NON27410 |  |  |
|  | f05 | November 2015 | Marseille | KOLMI M52000 |  |  |
|  | g01 | August 2015 | Marseille | SANDEL 15051876LB |  |  |
|  | g03 | May 2015 | Marseille | SANDEL 14121705LB |  |  |
|  | g09 | August 2015 | Marseille | KC AM5087421 |  |  |
|  | h01 | August 2015 | Marseille | KC AM4059361 |  |  |
|  | h02 | August 2015 | Marseille | KC AM5087421 |  |  |
|  | h07 | November 2015 | Marseille | SANDEL 85001 |  |  |
|  | h08 | August 2015 | Marseille | SANDEL 15051876LB |  |  |
|  | h10 | August 2015 | Marseille | KC AM4140362 |  |  |
|  | i2 | November 2015 | Marseille | ND |  |  |
|  | i4 | March 2016 | Marseille | ND |  |  |
|  | i5 | March 2016 | Marseille | ND |  |  |
|  | i6 | March 2016 | Marseille | ND |  |  |
|  | i7 | March 2016 | Marseille | ND |  |  |
|  | i8 | June 2016 | Marseille | ND |  |  |
|  |  |  |  |  |  |  |
|  | code | sampling date | city | sample localisation | hospitalisation service | medical history |
| PATIENTS | 1214 | August 2018 | Paris-PSL | mouth | Infectious and tropical diseases | heart transplant |
|  | 1239 | September 2018 | Paris-PSL | sputum | Pneumology | chronic obstructive pulmonary disease |
|  | 1252 | September 2018 | Paris-PSL | sputum | Pneumology | chronic obstructive pulmonary disease |
|  | 1272 | October 2018 | Paris-PSL | Tracheal aspiration | Medical intensive care | kidney transplant |
|  | 1275 | October 2018 | Paris-PSL | sputum | Pneumology | chronic obstructive pulmonary disease |
|  | 1276 | October 2018 | Paris-PSL | ear | Ear Nose and Throat | none |
|  | 1279 | October 2018 | Paris-PSL | ear | Ear Nose and Throat | none |
|  | 1282 | October 2018 | Paris-PSL | peritoneal membrane | Surgical intensive care | bariatric surgery |
|  | 1290 | November 2018 | Paris-PSL | ear | Ear Nose and Throat | none |
|  | 1299 | November 2018 | Paris-PSL | bronchoalveolar lavage | Pneumology | Carcinoma of the oesophagus |
|  | 1308 | December 2018 | Paris-PSL | ear | Ear Nose and Throat | chronic otitis |
|  | 1312 | December 2018 | Paris-PSL | cholesteatoma | Ear Nose and Throat | cholesteatome |
|  | 1325 | January 2019 | Paris-PSL | ear | Ear Nose and Throat | cholesteatoma and external otitis |
|  | 1328 | February 2019 | Paris-PSL | non bronchoscopic lavage | Pneumology | none |
|  | 1330 | January 2019 | Paris-PSL | pituitary adenoma | Diabetic medicine | none |
|  | 1356 | March 2019 | Paris-PSL | sputum | Pneumology | chronic obstructive pulmonary disease |
|  | 1381 | May 2019 | Paris-PSL | ear | Ear Nose and Throat | cholesteatoma |
|  | 1387 | May 2019 | Paris-PSL | maxillary sinus | Ear Nose and Throat | none |
|  | 1406 | June 2019 | Paris-PSL | ear | Ear Nose and Throat | chronic otitis |
|  | 1415 | July 2019 | Paris-PSL | toe nail | Dermatology | none |
|  | 1424 | July 2019 | Paris-PSL | sputum | Pneumology | none |
|  | 1436 | Septempber 2019 | Paris-PSL | sputum | Pneumology | chronic obstructive pulmonary disease |
|  | 1437 | Septempber 2019 | Paris-PSL | sputum | Pneumology | Mixed connective tissue disease |
|  | 1449 | Septempber 2019 | Paris-PSL | atrium | Cardiac Surgical Intensive Care | heart failure |
|  | 1457 | October 2019 | Paris-PSL | sputum | Pneumology | allergic bronchopulmonary aspergillosis |
|  | 1458 | October 2019 | Paris-PSL | ear | Ear Nose and Throat | external otitis |
|  | BDX-NC03 | March 2018 | Bordeaux | clinical ND | ND | ND |
|  | BDX-NC04 | March 2018 | Bordeaux | clinical ND | ND | ND |
|  | MTP-NC07 | April 2018 | Montpellier | clinical ND | ND | ND |
|  | MTP-NC08 | April 2018 | Montpellier | clinical ND | ND | ND |
|  | TLS-NC03 | March 2018 | Toulouse | clinical ND | ND | ND |
|  | TLS-NC04 | March 2018 | Toulouse | clinical ND | ND | ND |

Supplemental figure 1: comparison of the same deposit acquired on the three different instruments used for this study.


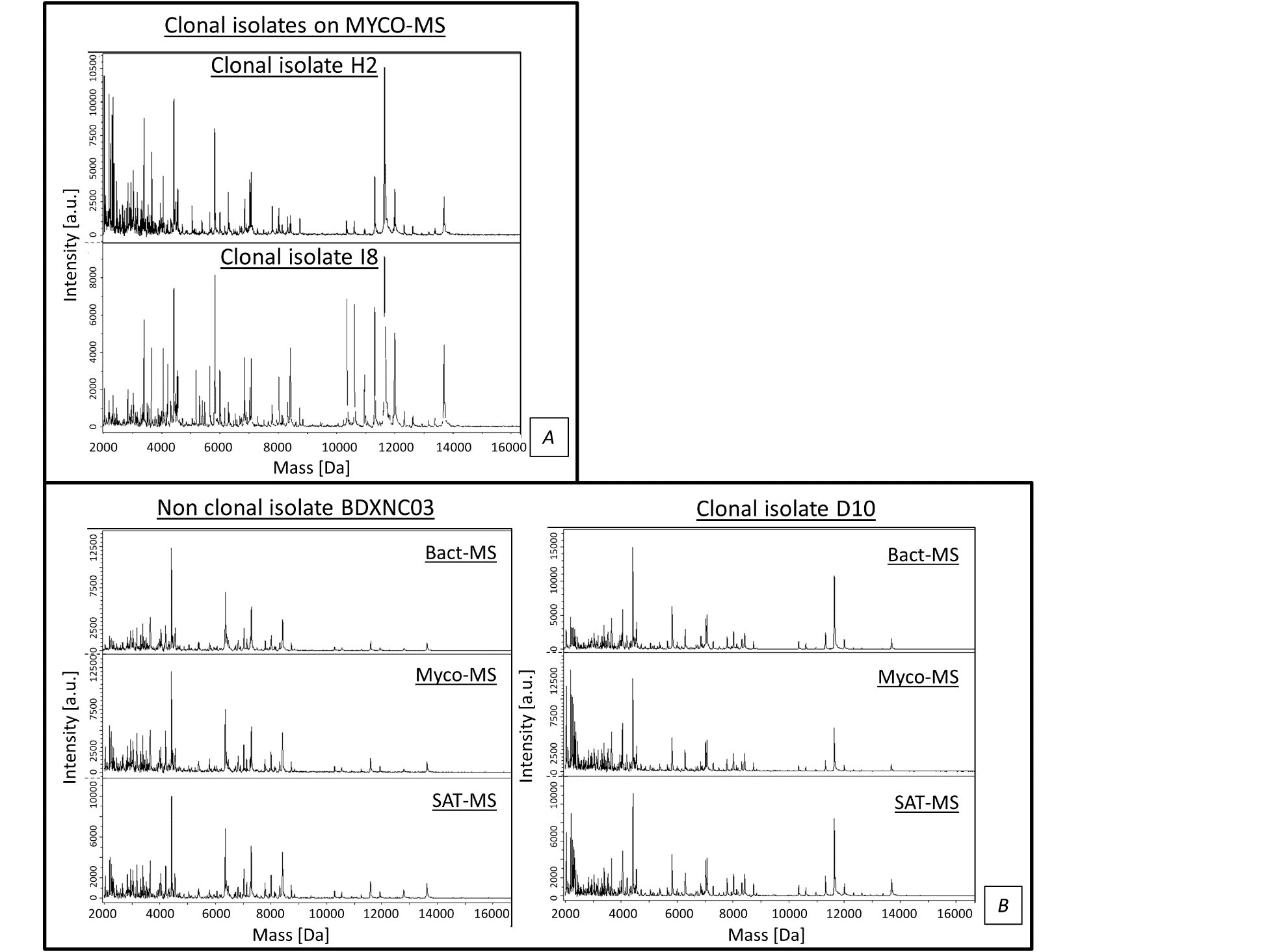

Supplement: Supplementary file 1 — Supplementary Information. [file 41598_2022_5647_MOESM1_ESM.docx]
